# Clinical staging and genetic profiling of Korean patients with primary lymphedema using targeted gene sequencing

**DOI:** 10.1038/s41598-022-17958-7

**Published:** 2022-08-10

**Authors:** Soo Hyun Seo, Seungjun Lee, Joseph Kyu-hyung Park, Eun Joo Yang, Boram Kim, Jee-Soo Lee, Man Jin Kim, Sung Sup Park, Moon-Woo Seong, Sun-Young Nam, Chan-Yeong Heo, Yujin Myung

**Affiliations:** 1 Department of Laboratory Medicine, Seoul National University Bundang Hospital, Seoul National University College of Medicine, Seoul, South Korea; 2 Department of Plastic and Reconstructive Surgery, Seoul National University Bundang Hospital, Seoul National University College of Medicine, Seoul, South Korea; 3 Department of Rehabilitation Medicine, Seoul National University Bundang Hospital, Seoul National University College of Medicine, Seoul, South Korea; 4 Department of Laboratory Medicine, Seoul National University Hospital, Seoul National University College of Medicine, Seoul, South Korea; 5 Biomedical Research Institute, Seoul National University Hospital, Seoul, South Korea

**Keywords:** Genetics, Physiology, Biomarkers, Pathogenesis

## Abstract

Lymphedema is a progressive disease caused by lymphatic flow blockage in the lymphatic pathway. Primary (hereditary) lymphedema is caused by genetic mutations without secondary causes. We performed clinical profiling on Korean primary lymphedema patients based on their phenotypes using lymphoscintigraphy and made genetic diagnoses using a next-generation sequencing panel consisting of 60 genes known to be related to primary lymphedema and vascular anomalies. Of 27 patients included in this study, 14.8% of the patients had lymphedema of the upper extremities, 77.8% had lymphedema of the lower extremities and 7.4% had 4-limbs lymphedema. Based on the International Society of Lymphology staging, 14, 10, and 3 patients had stage 3, 2, and 1 lymphedema, respectively. Only one family was genetically confirmed to harbor likely pathogenic variants in *CELSR1.* The proband was carrying two likely pathogenic variants in *CELSR1*, while her symptomatic mother was confirmed to carry only one of the variants. Furthermore, two other variants of uncertain significance in *CELSR1* were detected in other patients, making *CELSR1* the most commonly altered gene in our study. The clinical and genetic profile of hereditary lymphedema reported here is the first such data series reported for South Korea.

## Introduction

Lymphedema is a disorder of lymphatic transport caused by blockage and loss of function of the lymphatics. It is largely divided into primary lymphedema with no specific or unknown cause and secondary lymphedema with causes such as cancer-related treatment or filariasis. Primary lymphedema is further divided into types I and II according to the age of onset of symptoms^1^. Compared with that of secondary lymphedema, the prevalence of primary lymphedema is unknown. Although previous studies have reported a prevalence of approximately 1:100,000–500,000^2,3^, the actual global prevalence is estimated to be greater^4^. Primary lymphedema is often underdiagnosed as patients do not receive an accurate diagnosis of the actual cause of edema and are thereby subjected to various alternative tests and treatments owing to lack of accurate information about primary lymphedema^5^.

Type I (congenital) and type II (pubertal onset) primary lymphedema were first described by Milroy^2,3^ and Meige^4^ in 1892 and 1898, respectively. However, to date, no large-cohort study, including that on clinical phenotype patterns and genetic mutations involved, has been conducted on the East Asian primary lymphedema population. Therefore, in the present study, we aimed to perform accurate clinical profiling of Korean patients with primary lymphedema and to analyze their genetic variance using targeted gene sequencing. The gene sequencing study performed herein was part of a nationwide genetic study for the detection of rare diseases, which was sponsored by the South Korean national government (National Supporting Program for Genetic Diagnosis of Rare Diseases of the Korea Centers for Disease Control & Prevention).

## Results

### Clinical characteristics of patients

Of 27 consecutive primary lymphedema patients included in the study, 18 were female and 9 were male. Their mean age was 35 years (range, 1–77 years), and their average body mass index was 24.8. Notable past medical histories of the patients included ongoing lung cancer (n = 1), hypertension (n = 4), and diabetes mellitus (n = 2). Three patients had familial history of primary lymphedema in second-degree relatives. One proband and her mother were two of the three patients with a familial history. In 44.4% of the patients (n = 12), symptoms first appeared between the age of 20 and 40 years. In contrast, the rate of onset of symptoms before 10 years of age was 14.8% (n = 4), that between the ages of 10 and 20 years was 14.8% (n = 4), and that after age 40 was 25.9% (n = 7). The most common lymphedema location was unilateral lower extremity (n = 18, 66.7%), followed by bilateral lower extremity (n = 3, 11.1%), unilateral upper extremity (n = 3, 11.1%), bilateral upper extremity (n = 1, 3.7%), bilateral upper and lower extremity (n = 1, 3.7%) and bilateral arm, leg and face (n = 1, 3.7%).

**Figure 1:**
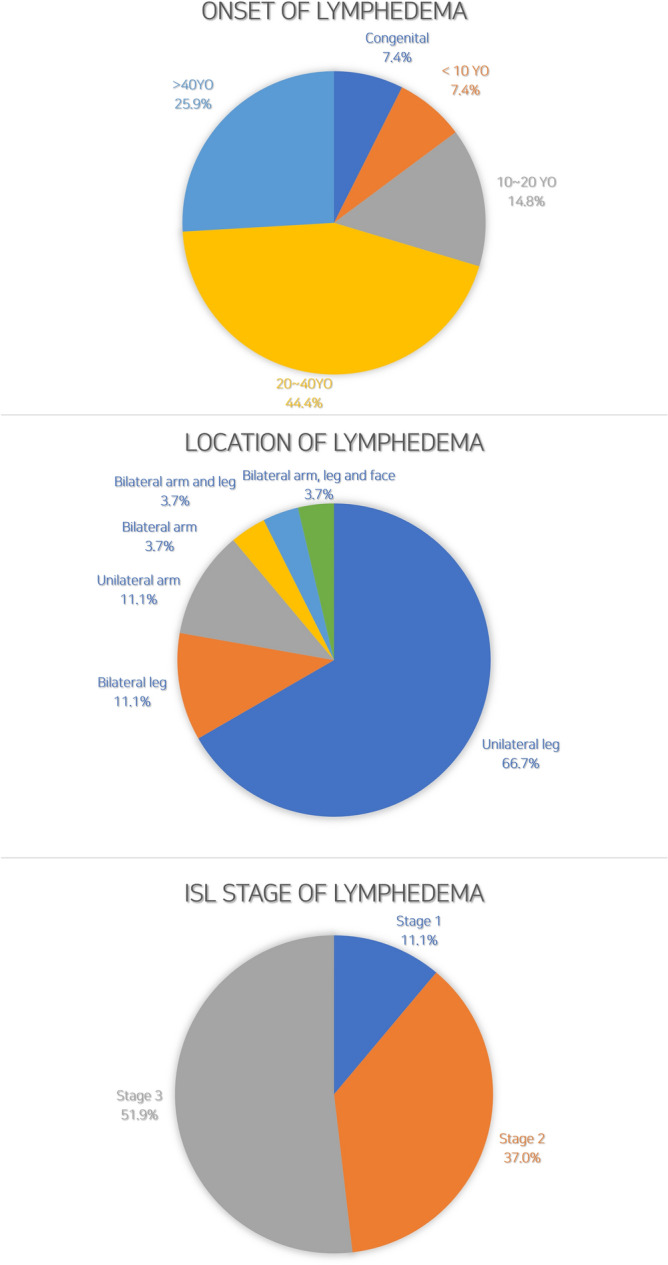
Onset (above), location (middle), and stages (below) of primary lymphedema in the patient cohort.

Based on the International Society of Lymphology (ISL) stages, stage 3 lymphedema was the most common (n = 14, 51.9%), followed by stage 2 (n = 10, 37.0%) and stage 1 (n = 3, 11.1%). The average difference in volume between the involved and normal limbs was 19% for lower-limb lymphedema (average 254 ± 35 cc) and 11% for upper-limb lymphedema (average 68 ± 12 cc). On lymphoscintigraphy images, apparent dermal backflow with or without axillary/inguinal lymph node uptake was observed in all patients, and in the case of lymphoscintigraphy grade, most patients (n = 19) showed total lymphatic obstruction (stage 4–6) in the involved limb. Indocyanine-green (ICG) lymphography was also performed in all the patients, and with respect to dermal backflow stages (0–V), most patients showed a stardust/diffuse pattern in majority of the involved limbs at stages IV (n = 15) and V (n = 7) (Fig. 1, Table 1).

**Table 1:** Patient demographics and phenotypic classifications.

	Number	Percentage (%)
Patients	27	
**Sex**		
Male	9	33.3
Female	18	66.7
Age (range)	35.6 ± 27.8 (1–77)	
**Initial symptom age**		
Congenital	2	7.4
< 10 years	2	7.4
10–20 years	4	14.8
20–40 years	12	44.4
> 40 years	7	25.9
**Location**		
Unilateral leg	18	66.7
Bilateral leg	3	11.1
Unilateral arm	3	11.1
Bilateral arm	1	3.7
4-limbs lymphedema	1	3.7
4-limbs and face	1	3.7
**Lymphedema stage**		
1	3	11.1
2	10	37.0
3	14	51.9
**Cellulitis history**		
Yes	12	44.4
No	15	55.6

### Germline variants of lymphedema-related genes

The average coverage depth in the target regions of the gene sequencing panel was 160.7X. On average, 92.8% bases had coverage ≥ 10X (45.2–99.5%). Among the 27 patients, only two were detected with pathogenic variants, and both belonged to the same family (Table 2). The proband was carrying two likely pathogenic variants in *CELSR1*, while her symptomatic mother was confirmed to carry only one variant. In addition, two other variants of uncertain significance (VUSs) were detected in *CELSR1* in other patients, making *CELSR1* the most commonly altered gene in our study.

**Table 2:** Genetic and clinical profiles of 4 patients with genetic abnormalities detected in *CELSR1*. All 4 variants were not found in the general population (gnomAD).

Case	Sex/age	Gene	Transcript	DNA variants	Protein alteration	Zygosity	Classification	Clinical staging	Location of edema
1	39/F	*CELSR1*	NM_014246.3	c.8446C>T	p.Gln2816*	Heterozygous	Likely pathogenic	3	Bilateral arm and leg
		*CELSR1*	NM_014246.3	c.8871_8872del	p.Cys2957*	Heterozygous	Likely pathogenic	3	Bilateral arm and leg
2*	67/F	*CELSR1*	NM_014246.3	c.8446C>T	p.Gln2816*	Heterozygous	Likely pathogenic	1	Bilateral arm and leg
3	30/M	*CELSR1*	NM_014246.3	c.2017G>A	p.Val673Met	Heterozygous	VUS	3	Left leg
6	27/F	*CELSR1*	NM_014246.3	c.5642G>A	p.Cys1881Tyr	Heterozygous	VUS	3	Left leg

*VUS* variant of uncertain significance, *gnomAD* Genome Aggregation Database.

*Case 2 is mother of case 1.

A nonsense variant of *CELSR1*, c.8446C>T (p.Gln2816*) and a frameshift variant, c.8871_8872del (p.Cys2957*) were detected in the patient presented as case 1 (Table 2). c.8446C>T, which was classified as a likely pathogenic variant, was also detected in the patient’s symptomatic mother who was included in the study group. The second variant, c.8871_8872del, was also classified as a likely pathogenic variant; however, this variant was not detected in the patient’s mother. Her asymptomatic father was presumed to be a carrier of the variant, but it could not be confirmed.

## Discussion

In the present study, we analyzed the clinical characteristics and genetic profiles of 27 patients with primary lymphedema. To the best of our knowledge, this is the first clinical and genetic profiling study on a primary lymphedema cohort from a single institution in East Asia.

To date, studies on primary lymphedema have mainly focused on reviewing clinical features^6^ of patients and discussing relevant medical and surgical treatments. Since the features of this disease were first described by Milroy and Meige, sporadic presentations of different phenotypical patterns have been reported^7–12^; accordingly, the disease pattern and diagnostic criteria have changed over the years^1,3,6,13,14^. There have been numerous approaches to identify causative genes or loci responsible for primary lymphedema or associated complicated lymphatic anomalies^15–18^.

Among the 27 primary lymphedema patients included in this study, the family of only one patient was genetically confirmed to have a likely pathogenic variant of *CELSR1*, which has been previously reported as causative for nonsyndromic hereditary lymphedema. In 2016, Gonzalez-Garay et al.^19^ reported a family with symptomatic hereditary lymphedema across three generations based on a proband with a mutation in *CELSR1*. In 2019, Erickson et al.^20^ reported a family with *CELSR1* haploinsufficiency, where lymphedema affected only the female members and the patients presented with lymphangiectasia, valve dysfunction, and thoracic duct reflux. Moreover, in 2019, Maltese et al.^21^ reported that 5 of 95 probands carried novel loss-of-function variants in *CELSR1.* In 2021, Xia et al.^22^ reported that a *CELSR1* deletion could be associated with lymphatic dysplasia in a lymphedema patient with 22q13.3 deletion syndrome. In the case with two likely pathogenic variants of *CELSR1* reported here, the proband exhibited a more severe phenotype compared with her mother, who carried only one of the variants. Although no other female family member in this family was confirmed to carry only the second frameshift variant, it appears that carrying the additional likely pathogenic variant leads to the more severe phenotype.

Morphological diagnosis of edema; functional imaging, including lymphoscintigraphy and ICG lymphangiogram; and in particular, the absence of secondary etiologies such as cancer-related treatment, parasitic infections^23,24^ (e.g., filariasis), and trauma are important for the diagnosis of primary lymphedema. In the case of primary lymphedema of unknown etiology, there is very little information on disease progression, severity, and treatment strategies. However, as in the case presented in this study, early genetic detection in patients with nonsyndromic primary lymphedema enables accurate diagnosis of lymphedema and prompt intervention, such as comprehensive rehabilitation and supermicrosurgical treatment, in earlier stages to prevent disease progression, which have been showing good results in the primary lymphedema population^25^. Additional case reports and genotype–phenotype information are needed to develop a more fundamental approach to treat primary lymphedema, which is still lacking.

The main limitation of the present study is the small sample size. The study cohort was limited to patients with primary lymphedema who visited a single institution for a certain period. However, given that primary lymphedema is an uncommon disease, it is difficult to perform studies on a large cohort. Studies involving not only Korean patients but also patients of other ethnicities are needed to gain accurate and detailed information about primary lymphedema. Additionally, the detection rate of mutations was not very high in this study, although VUSs need to be evaluated further. Sufficient information is not available on causative genetic variants for primary lymphedema, warranting additional studies investigating the involvement of unknown candidate genes. In addition, although the samples and targeted gene sequencing results indicated novel findings regarding the Asian primary lymphedema population, owing to the limitations of performing a nationwide genetic study, there remain other genes that should be investigated for pathogenic variants^17^. Variants in these genes can account for cases of primary lymphedema, and these genes must be investigated in the near future.

In conclusion, this study highlights the heterogenic nature of primary lymphedema and bridges the knowledge gap in genetic profiling of primary lymphedema in Korean patients. In addition, we confirm the diagnostic utility of gene sequencing in primary lymphedema. We also report the possibility that a variant in *CELSR1*, which to the best of our knowledge has not been reported earlier in this population, is a cause of nonsyndromic primary lymphedema in Koreans and East Asians. These results confirm the importance of genetic testing for primary lymphedema and highlight the need for further investigations of the use of targeted gene sequencing as an important tool for the diagnosis of primary lymphedema, facilitating early medical intervention.

## Methods

### Patients

Primary lymphedema patients who visited Hereditary Lymphedema Clinic, Seoul National University Bundang Hospital (Seongnam, South Korea) between February 2020 and October 2021 were included in the present study. Primary lymphedema was diagnosed on the basis of symptoms, physical examination, patient history, and imaging studies. All patients underwent clinical examination by a rehabilitation specialist (EJY) and a lymphedema microsurgeon (YJM). Written informed consent was obtained from all patients. This study was performed in accordance with the Declaration of Helsinki (2013) and was approved by the Institutional Review Board of Seoul National University Bundang Hospital (B-2112-724-103).

### Clinical profiling

#### Clinical diagnosis of lymphedema

Clinical diagnosis of lymphedema was made based on the location and severity of the disease. Lymphedema staging was performed based on the ISL staging criteria^26^. If the stage of each part of the involved limb was different, the staging was based on the site with the most severe lymphedema.

Medical history and onset and duration of lymphedema were recorded for each patient. The types of treatment that the patient had undergone previously were classified. In addition, the possibility of syndromic appearance other than lymphatic malformation was investigated to rule out other anomalies and diseases. Finally, patient family history was investigated, and patients with family members or close relatives with symptomatic lymphedema were noted.

### Volume estimation

Preoperative limb circumferences were measured using tape measures. For the arms, measurements were made 10 cm above, at, 10 cm below, and 20 cm below the elbow. For the legs, measurements were made 20 cm above, 10 cm above, at, 10 cm below, and 20 cm below the knee. The volumes of the limbs were estimated using the formula of a truncated cone.

### Targeted Gene Sequencing

DNA was extracted from whole blood samples collected from the 27 patients. Targeted gene sequencing was performed on DNA samples enriched using the SureSelectXT Human all Exon 50 Mb kit (Agilent, Santa Clara, CA, USA) on the Illumina HiSeq sequencing system (Illumina, Inc., San Diego, CA, USA) with 100-bp paired end reads. Sequencing alignment and variant calling were performed using NextGENe software (SoftGenetics, State College, PA, USA).

### Variant filtering and interpretation of clinical significance

Variants were screened for the following 60 genes that are known to be related to primary lymphedema/vascular anomalies: *ACVRL1*, *AKT1*, *ARAF*, *ARHGAP31*, *BRAF*, *CBL*, *CCBE1*, *CCM2*, *CELSR1*, *CTNNB1*, *DCHS1*, *ELMO2*, *ENG*, *EPHB4*, *FAT4*, *FGFR1*, *FLT4*, *FOXC2*, *GATA2*, *GDF2*, *GJC2*, *GLMN*, *GNA11*, *GNA14*, *GNAQ*, *HGF*, *HRAS*, *IDH1*, *IDH2*, *KIF11*, *KRAS*, *KRIT1*, *MAP2K1*, *MAP2K2*, *MAP3K1*, *MAP3K3*, *MAPK1*, *MAPK14*, *MAPK3*, *MET*, *MTOR*, *NRAS*, *PDCD10*, *PDGFRB*, *PIEZO1*, *PIK3CA*, *PTEN*, *PTPN11*, *PTPN14*, *RAF1*, *RASA1*, *RIT1*, *SHOC2*, *SMAD4*, *SOS1*, *SOX18*, *STAMBP*, *TEK*, *TP53*, and *VEGFC*. Exonic variants including nonsynonymous variants and intronic variants within 10 bp from the exonic region were included in the analysis. Classification of each retained variant was performed according to the American College of Medical Genetics and Genomics and the Association for Molecular Pathology guidelines^27^.

## Data Availability

The data that support the findings of this study are available from Seoul National University Bundang Hospital, but restrictions apply to the availability of these data, which were used under license for the current study, and are therefore not publicly available. Data are however available from the authors upon reasonable request and with permission of the corresponding author and the Institutional Review Board of Seoul National University Bundang Hospital.
